# Improving a Bimanual Motor Skill Through Unimanual Training

**DOI:** 10.3389/fnint.2016.00025

**Published:** 2016-07-14

**Authors:** Takuji Hayashi, Daichi Nozaki

**Affiliations:** ^1^Division of Physical and Health Education, Graduate School of Education, The University of TokyoTokyo, Japan; ^2^Japan Society for the Promotion of ScienceTokyo, Japan; ^3^Center for Barrier-Free Education, Graduate School of Education, The University of TokyoTokyo, Japan

**Keywords:** unimanual movement, bimanual movement, reaching movement, context-dependent motor memories, motor adaptation, learning transfer, whole and part practice

## Abstract

When we learn a bimanual motor skill (e.g., rowing a boat), we often break it down into unimanual practices (e.g., a rowing drill with the left or right arm). Such unimanual practice is thought to be useful for learning bimanual motor skills efficiently because the learner can concentrate on learning to perform a simpler component. However, it is not so straightforward to assume that unimanual training (UT) improves bimanual performance. We have previously demonstrated that motor memories for reaching movements consist of three different parts: unimanual-specific, bimanual-specific, and overlapping parts. According to this scheme, UT appears to be less effective, as its training effect is only partially transferred to the same limb for bimanual movement. In the present study, counter-intuitively, we demonstrate that, even after the bimanual skill is almost fully learned by means of bimanual training (BT), additional UT could further improve bimanual skill. We hypothesized that this effect occurs because UT increases the memory content in the overlapping part, which might contribute to an increase in the memory for bimanual movement. To test this hypothesis, we examined whether the UT performed after sufficient BT could improve the bimanual performance. Participants practiced performing bimanual reaching movements (BM) in the presence of a novel force-field imposed only on their left arm. As an index for the motor performance, we used the error-clamp method (i.e., after-effect of the left arm) to evaluate the force output to compensate for the force-field during the reaching movement. After sufficient BT, the training effect reached a plateau. However, UT performed subsequently improved the bimanual performance significantly. In contrast, when the same amount of BT was continued, the bimanual performance remained unchanged, highlighting the beneficial effect of UT on bimanual performance. Considering memory structure, we also expected that BT could improve unimanual performance, which was confirmed by another experiment. These results provide a new interpretation of why UT was useful for improving a bimanual skill, and propose a practical strategy for enhancing performance by performing training in various contexts.

## Introduction

**Figure 1:**
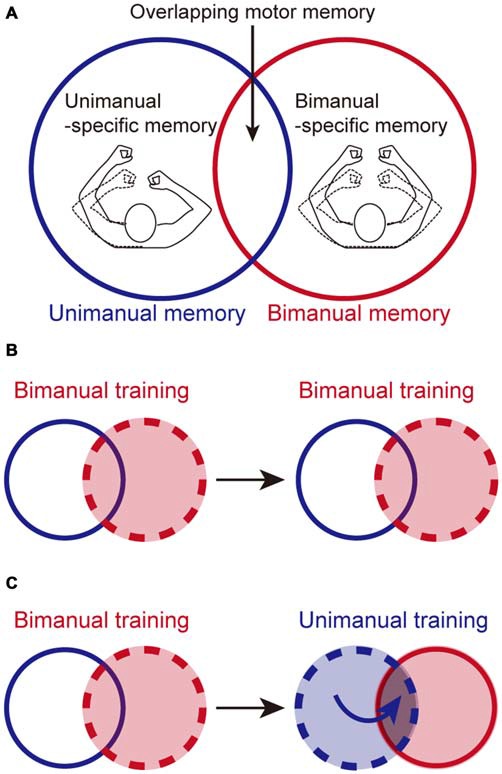
**Possible beneficial effect of unimanual training (UT) on bimanual performance, based on partially segregated motor memories. (A)** Motor memories for identical reaching movements consist of three distinct parts: unimanual-specific, bimanual-specific, and overlapping parts, as we reported previously (Nozaki et al., 2006; Nozaki and Scott, 2009). **(B)** Bimanual training (BT) effects should be stored in the bimanual-related parts (i.e., bimanual-specific and overlapping part). Once the memory content reached a plateau, it is not possible to increase the memory content by performing additional BT. **(C)** However, performing additional UT could enhance bimanual performance by adding training effects to the overlapping part of the memory structure. Broken circles in **(B,C)** represent the training contexts.

When we try to learn a complicated motor skill, we often break it down into its simpler fundamental skills (Part practice: Schmidt and Wrisberg, 2007; Schmidt and Lee, 2011). One of the representative examples is bimanual skills. In the case of rowing a boat, for example, it is a common practice to pull an oar with each arm separately before rowing with both arms together (McArthur, 1997). Practically, this type of training is beneficial, because a single yet complicated unimanual skill can be trained first, before performing the same action bimanually (Schmidt and Wrisberg, 2007). However, it is not as straightforward as assuming that unimanual training (UT) improves bimanual performance. We have demonstrated that the adaptation of reaching movements to a novel force-field environment is only partially transferred to the same arm movement when the movement of the opposite arm is absent (i.e., a unimanual reaching movement: UM) or present (i.e., a bimanual reaching movement: BM; Nozaki et al., 2006; Nozaki and Scott, 2009; Kadota et al., 2014). From this observation, we proposed that the motor memories for identical movements are partially segregated: UM-specific, BM-specific, and overlapping parts (Figure 1A). Such a memory structure would explain why motor adaptation is only partially transferred between unimanual and bimanual movement. Recent studies have shown that the motor memories for an identical movement can be flexibly switched according to different behavioral contexts, such as how the opposite arm is moving (Howard et al., 2010; Yokoi et al., 2011, 2014), whether the movement is performed discretely or rhythmically (Ikegami et al., 2010; Howard et al., 2011), and what kind of movement followed afterwards (Howard et al., 2015).

This memory structure suggests that UM training is not particularly effective for improving BM skills, because its training effect would be transferred only partially to BM. Counter-intuitively, however, we speculated that UM training could improve BM performance based on this memory structure. Consider the case when the adaptation of the left arm to a certain level of force-field is achieved by BM training. The adaptation effect is stored in the BM memory (i.e., BM-specific and overlapping parts; Figure 1A). Once the training effect is virtually saturated, the amount of BM memory cannot be increased any further by performing additional BM training trials (Figure 1B). It should be noted that Figure 1B does not mean that the memory is full, but that the amount of BM memory has reached the upper limit that can be learned through the training. For example, if the force-field level is further increased, the movement error resulting from the increase can increase the amount of BM memory. However, this is not the only way to increase the amount of BM memory. Even if the force-field level is maintained, the amount of BM memory can be also improved by performing UM training, as described below. Since the total amount stored in UM memory (i.e., UM-specific and overlapping components) is not yet saturated, performing UM could lead to movement error. This movement error could increase the total amount of UM memory (Figure 1C). Since this increment is accompanied by an increase in memory in the overlapping part, the total amount of motor BM memory (i.e., BM-specific and overlapping parts) is further increased (Figure 1C), which can lead to improvement in BM performance. In other words, the UM training may bring a breakthrough effect over that achieved by BM training. In order to test this hypothesis, we used an experimental paradigm of motor adaptation to a novel force-field during a reaching movement to investigate whether performing UM training after BM training could improve BM performance more than continuing to perform only BM training could. Considering that the memory structure is almost symmetrical (Figure 1A), we predicted that the same effect should be also observed for the UM performance as when additional BM training was performed, and tested this hypothesis.

## Materials and Methods

### Participants

Fifty-two right-handed participants (35 male, 17 female, age: 19–52 years) were recruited in the following experiments. The participants had no reported cognitive, motor, or neurological disorders. We obtained written informed consent from all participants prior to commencing experiments. The Ethics Committee of the University of Tokyo reviewed and approved the experimental protocol that was in accordance with the Declaration of Helsinki.

### Apparatus and Motor Tasks

**Figure 2:**
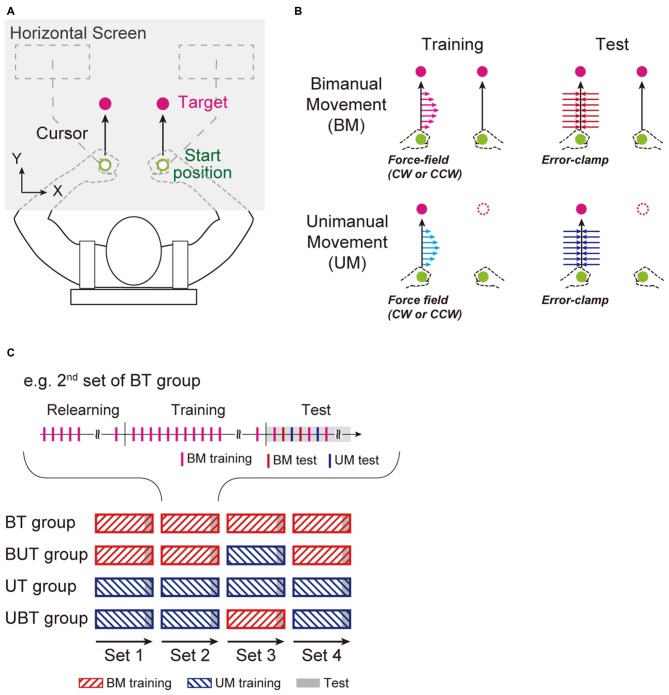
**Experimental setup. (A)** Right-handed participants performed left arm reaching movement without (unimanual reaching movement: UM), or with, simultaneous right hand movement (bimanual reaching movement: BM) to move cursors (white circles) representing the handle position from the start positions (green circles) to targets (magenta circles). The participants were instructed to move the handles as straight as possible (black arrows). **(B)** Four types of trials were performed: 2 task types [Training (force-fields: left column) and Test (error-clamp: right column)] × 2 movement types [BM (upper row) and UM (bottom row)]. **(C)** There were four experimental groups (BT, Bimanual-Unimanual Training [BUT], UT, and Unimanual and Bimanual training [UBT] groups). In the BT group, four BM training sets (bars with red hatched lines) were performed (each set consisted of 64 training trials), and in the test period at the end of each set (gray-shaded area), the training effects on BM and UM performance were evaluated by pseudo-randomly interleaving BM and UM test (10 error-clamp trials for each movement). At the beginning of each set, the participants performed 10 training trials in the same training context as used in the previous set, so that the training effect could be recovered after a short break (2–3 min). In the BUT group, the 3rd training set was replaced by UM training. The procedures were identical for the UT and UBT groups.

The participants performed horizontal UM and BM holding robotic handles (KINARM End-Point lab, Bkin Technologies, Kingston, ON, Canada; Figure 2A). They sat in an adjustable chair to which their back was strapped. Their wrists were constrained by braces so that unnecessary wrist movements did not occur. They were instructed to move white cursors (diameter: 10 mm) representing the hand positions, from start positions (diameter: 14 mm) to targets (diameter: 14 mm) on a horizontal display. They could not directly see the movement of their arms. The start positions were located at approximately 15 cm in front of their body and the distance between the positions for both arms was 15 cm. The targets were located 10 cm straight ahead of the start positions. To begin each trial, the participants were required to move and maintain the cursor at the start positions for 1 s. A green target appeared and then turned magenta after additional 1–1.5 s holding times, indicating the “go” cue. The participants were instructed to perform the UM with the left arm or to perform BM toward the targets, as straight as possible, and to focus on the left hand movements even during the BM condition. Warning messages were presented immediately below the start positions if the peak movement velocity was below (“slow”) or above (“fast”) a range (340–460 mm/s). After completion of each trial, the handles were automatically moved back to the start positions without the participant’s efforts.

For the training, the participants performed reaching movements (UM or BM) under the presence of a velocity-dependent curl force-field (imposed only on the left arm) as *f* = *Bν* (Shadmehr and Mussa-Ivaldi, 1994; Figure 2B), where *f* = [*f_x_*; *f_y_*] (*N*) is the force to the left handle, *ν* = [*ν_x_*; *ν_y_*] (m/s) is the left handle velocity, *B* = [*0, −b; b, 0*] is the viscosity matrix, and *b* (N/[m/s]) is the viscosity. Thus, in this study, the left hand movement was trained by the force-field for both UM and BM. Half of the participants adapted to a clockwise force-field (*b* = −15) and the other participants adapted to a counter-clockwise force-field (*b* = −15). The right hand movements during BM were not perturbed throughout the experiment.

To evaluate the effect of training of the left arm (i.e., the level of adaptation), we used the “error-clamp method” with which the movement trajectory of the left handle was constrained to a straight path from the start position to the target by a virtual force-channel (Scheidt et al., 2000; Figure 2B). The force-channel was created by a virtual spring (6000 N/m) and dumper (100 N/[m/s]) in the perpendicular direction to the straight path of movement. This method enabled us to measure lateral force output exerted against the channel (i.e., the after-effect) directly. Error-clamp trials were not applied to the right arm throughout the experiments.

### Experimental Groups and Flows

There were four experimental groups, determined according to the training protocols assigned (Figure 2C). In one of the groups (Bimanual Training (BT), *n* = 12), after 10 BM trials in the null force-field condition, participants performed BM training for four consecutive sets. In each set, participants performed 64 BM training trials in the training period (Figure 2C). This training period was followed by a testing period in which 10 BM training, 10 BM error-clamp trials, and 10 UM error-clamp trials were pseudo-randomly interleaved, to evaluate the level of motor adaptation for BM and UM. Since such breaks would decay the level of adaptation to the force-field acquired by the previous training set, the participants performed 10 training trials used in the previous set (in this case, BM training) at the beginning of each set (the 2nd, 3rd, and 4th set), to recover the adaptation level again to the level before the break. To test the hypothesis that UM training enhanced BM performance, in another experimental group (Bimanual-Unimanual Training (BUT) group; *n* = 12), the 3rd training set was replaced by UM training trials (Figure 2C). That is, the testing period in the BUT group included 10 UM training trials, and 10 BM and 10 UM error-clamp trials. We compared the BM performance at the end of the 3rd set between the BT and BUT groups. If the BM performance was greater for the BUT group than for the BT group, we could conclude that UM training had a beneficial effect on BM performance.

We also examined whether BM training enhanced UM performance by testing the UT and UBT group (*n* = 14 for each group). The UT group performed four consecutive UM training sets, while the UBT group trained with BM training in the 3rd set (Figure 2C). The settings of the training period, testing period, and short break were identical to those of the BT and BUT groups.

### Data Analysis

The handle positions and forces were sampled at 1000 Hz. The data regarding the handle positions were smoothed by a fourth-order Butterworth filter with a cutoff frequency of 10 Hz. To quantify kinematic errors in the training conditions, we measured the lateral deviation at the peak velocity of the handle, from the straight line between the start positions to the targets. A lateral deviation in the force-field direction was defined as positive. In test trials, we evaluated the after-effect for the adaptation level as *f_pv_/v_pv_*, where the *f_pν_* was the lateral force against the force-channel evaluated at the peak velocity *ν_pν_*. The *f_pν_* in the opposite direction of the force-field was defined as positive. A value of 15 (i.e., |*b*|) indicates that the adaptation level was 100%, whereas a value of 0 indicates no adaptation.

To examine how performing training trials with different movement patterns in the 3rd set changed the adaptation levels, we performed a 2-way repeated measures ANOVA (Groups and Sets) for each of the movement types (i.e., the UM and BM test trials). If a significant interaction and a simple main effect for groups were obtained, we continued to perform multiple comparisons, with Bonferroni correction as *post hoc* tests. The statistically significant threshold was set at *P* < 0.05.

## Results

**Figure 3:**
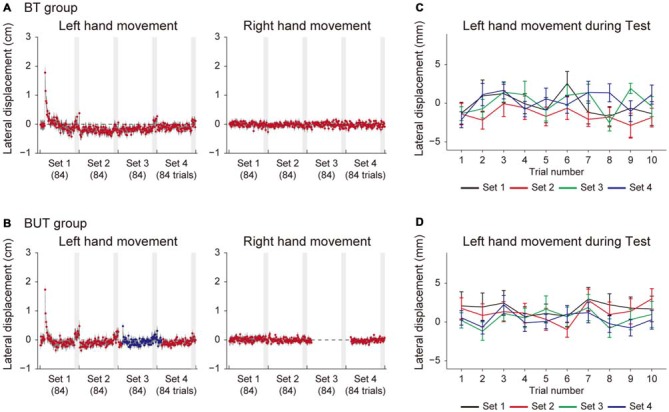
**Trial dependent change in the movement error for BT and BUT groups. (A,B)** Trial-dependent changes in the lateral deviation of the left hand (left panels) and right hand (right panels) for the BT group **(A)** and the BUT group **(B)**. The blue and red dots indicate lateral deviations at the peak velocities of UM and BM movements, respectively. Gray-shaded areas represent the test periods. **(C,D)** Trial-dependent changes in the lateral deviation of the left hand during the test period (10 trials) for the BT group **(C)** and BUT group **(D)**. All error bars indicate SEM.

Figure 3 shows the lateral deviations of both hands at the peak velocities in training trials for the BT group (Figure 3A) and the BUT group (Figure 3B). The kinematic errors of the left hand (the left panel of Figures 3A,B) were produced by the force-field in the initial few trials, but gradually reduced by the end of the 1st set. Note that, since the force-field was imposed only on the left hand, the right hand movements were not affected by it (the right panel of Figures 3A,B). In the 3rd set of the BUT group (Figure 3B), a slight increase in error was observed, because the training context switched from BM to UM. This result was consistent with a previous finding that the training effect of BM was only partially transferred to UM (Nozaki et al., 2006; Nozaki and Scott, 2009; Kadota et al., 2014).

At the end of each set, we interleaved 10 BM and 10 UM error-clamp trials to evaluate the level of adaption by the force output (i.e., the after-effect). During this training period, the movement error (i.e., lateral deviation) slightly increased in the training trials during the test period (Figures 3A,B), possibly due to the memory decay caused by the error-clamp trials. We examined whether the adaptation level was maintained throughout the test period. If the motor memory gradually decreased along with memory decay, the lateral deviation of the hand during the test period would also gradually increase. However, Figures 3C,D indicate there was no such systematic data trend in lateral deviation during the test period (10 training trials were performed for each set).

**Figure 4:**
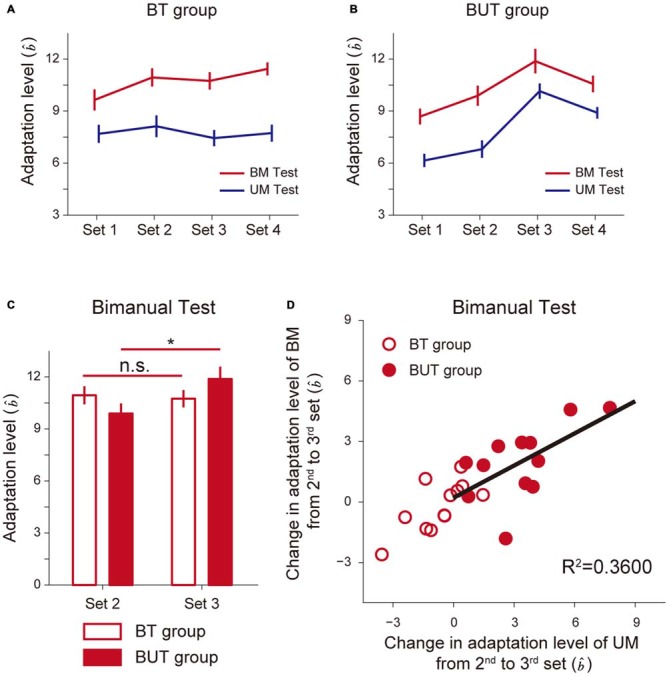
**Improvement of bimanual performance by UT. (A,B)** Adaptation levels were calculated by the ratio of the after-effect (generated force to the virtual force-channel) to the peak velocity for the BT group **(A)** and the BUT group **(B). (C)** The changes in the adaptation levels from 2nd to 3rd sets. *Indicates statistically significant differences in the levels between the 2nd and 3rd sets only for the BUT group (*p* < 0.05, Bonferroni correction). **(D)** A significant correlation was found between improvement in UM performance and that in BM performance. All error bars indicate SEM.

Figure 4 indicates how the adaptation level changed with sets for the BT group (Figure 4A) and the BUT group (Figure 4B). A 2-way repeated measures ANOVA applied to the adaptation level of BM revealed significant interactions between the BT and BUT groups (Groups and Sets: *F*_(1,3)_ = 5.39, *p* = 2.22 × 10^−3^, Figures 4A,B). There was no significant simple main effect of group for 2nd (*F*_(1,22)_ = 1.80, *p* = 0.19) and 3rd sets (*F*_(1,22)_ = 1.73, *p* = 0.20), but a significant simple main effect of sets was observed for both the BT group (*F*_(3,22)_ = 5.58, *p* = 5.30 × 10^−3^) and the BUT group (*F*_(3,22)_ = 17.37, *p* = 5.20 × 10^−6^). Multiple comparisons with Bonferroni corrections indicated that the adaptation level remained unchanged after the 2nd set for the BT group (2nd and 3rd set: *t*_(11)_ = 0.552, *p* > 1, corrected), implying that the adaptation level had almost reached a plateau (Figure 4C). In contrast, for the BUT group, the adaptation level was significantly increased from the 2nd to the 3rd set (*t*_(11)_ = 3.81, *p* = 0.017, corrected), indicating the beneficial effect of UM training on BM performance (Figure 4C). The adaptation level appears to decrease from the 3rd to the 4th set, but it was not statistically significant (*t*_(11)_ = 2.34, *p* = 0.24, corrected).

Of added note, since BT was conducted throughout all four sets, the UM adaptation level quantified by UM error-clamp trials was smaller than that for BM (Figures 4A,B), which was consistent with our previous finding of partial learning transfer from BM to UM skills (Nozaki et al., 2006; Nozaki and Scott, 2009). In the BUT group, the training in the 3rd set was performed unimanually, which increased the UM adaptation level from the 2nd to the 3rd set. A 2-way repeated measures ANOVA applied to the UM adaptation level revealed significant interactions between the BT and BUT groups (Groups and Sets: *F*_(1,3)_ = 22.64, *p* = 3.43 × 10^−10^, Figures 4A,B). There was a significant simple main effect of group, not for the 2nd set (*F*_(1,22)_ = 2.69, *p* = 0.12), but rather for the 3rd set (*F*_(1,22)_ = 17.70, *p* = 3.64 × 10^−4^). A significant simple main effect of sets was observed only for the BUT group (BUT group: *F*_(3,22)_ = 33.42, *p* = 2.27 × 10^−8^; BT group: *F*_(3,22)_ = 0.78, *p* = 0.52). Multiple comparisons with Bonferroni corrections indicated that the adaptation level remained unchanged after the 2nd set for the BT group (2nd and 3rd set: *t*_(11)_ = 1.74, *p* = 0.66, corrected; Figure 4A). In contrast, for the BUT group, the UM adaptation level significantly increased from the 2nd to the 3rd set (*t*_(11)_ = 5.67, *p* = 8.73 × 10^−4^, corrected).

Figure 4D indicates that there was a significant correlation between changes in BM and UM performance from the 2nd to the 3rd set (*R*^2^ = 0.3600, *t*_(10)_ = 2.372, *p* = 0.039), suggesting that the increase in the UM adaptation level (UM performance) might contribute to the increase in the BM adaptation level (BM performance), possibly through an increase in memory in the overlapping part of the motor memory structure (Figure 1C).

**Figure 5:**
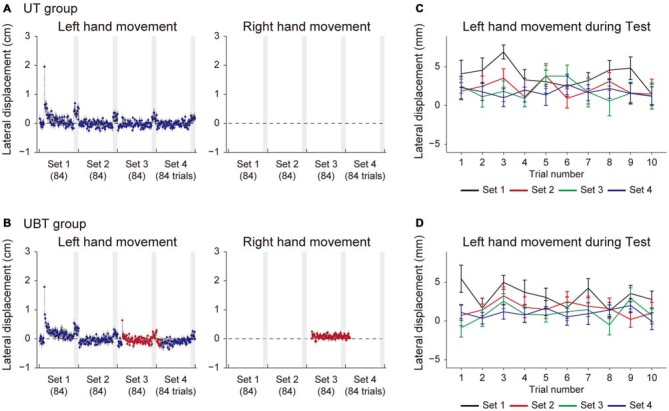
**Trial dependent change in the movement error for UT and UBT groups. (A,B)** Trial-dependent changes in the movement error of the left hand (left panels) and right hand (right panels) for the UT group **(A)** and the UBT group **(B)**. The blue and red dots indicate lateral deviations at the peak velocities of UM and BM movements, respectively. Gray-shaded areas represent the test period.** (C,D)** Trial-dependent changes in the lateral deviation of the left hand during the test period (10 trials) for the UT group **(C)** and the UBT group **(D)**. All error bars indicate SEM.

**Figure 6:**
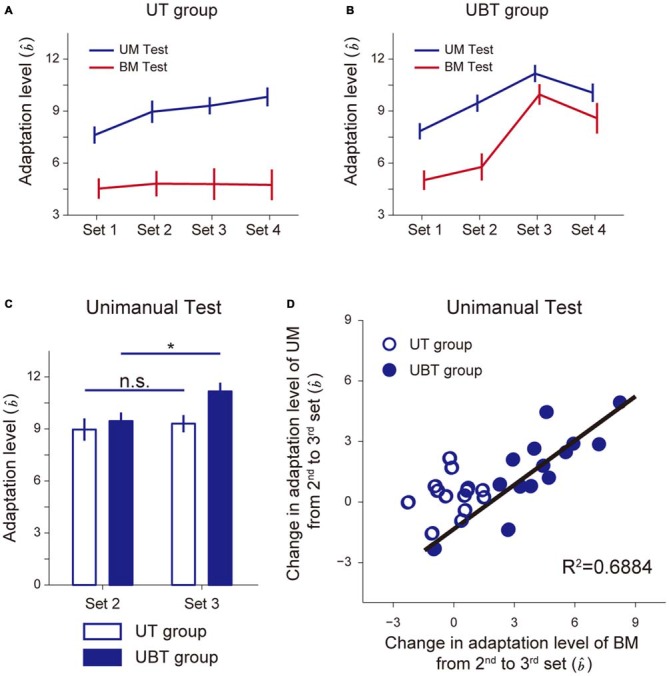
**Improvement of unimanual performance by BT. (A,B)** Adaptation levels were calculated by the ratio of the after-effect (the generated force to the virtual force-channel) to the peak velocity for the UT group **(A)** and the UBT group **(B). (C)** Changes in the adaptation levels from the 2nd to 3rd sets. *Indicates the statistically significant differences in the levels between the 2nd and 3rd sets only for the UBT group (*p* < 0.05, Bonferroni correction). **(D)** A significant correlation was found between improvement in BM performance and that in UM performance. All error bars indicate SEM.

Figures 5, 6 indicate the results for the UT and UBT groups. Essentially, the results were similar to those observed for the BT and BUT groups. The kinematic errors of the left hand (the left panel of 5A,B) were produced by the force-field in the initial few trials, but gradually reduced by the end of the 1st set. In the 3rd set of the UBT group (Figure 5B), a slight increase in error was observed, because the training context switched from UM to BM. Figures 5C,D indicate that there was no systematic trend in the lateral deviation of the hand during the test period, indicating that gradual memory decay during the test period did not occur. As for the level of motor adaptation, a 2-way repeated measures ANOVA revealed significant interactions for the adaptation levels of UM between the UT and UBT groups (Groups and Sets: *F*_(1,3)_ = 2.94, *p* = 0.038, Figures 6A,B). There was a significant simple main effect of group, not for the 2nd set (*F*_(1,26)_ = 0.371, *p* = 0.55), but rather for the 3rd set (*F*_(1,26)_ = 6.98, *p* = 0.013), and a simple main effect of sets was also significant for both groups (UT group: *F*_(3,26)_ = 7.46, *p* = 9.25 × 10^−4^; UBT group: *F*_(3,26)_ = 16.21, *p* = 3.83 × 10^−8^). Multiple comparisons with Bonferroni corrections revealed that the adaptation level in the UT group remained unchanged after the 2nd set (2nd and 3rd set: *t*_(13)_ = 1.36, *p* > 1, corrected; 3rd and 4th set: *t*_(13)_ = 1.51, *p* = 0.93, corrected; Figure 6C), indicating that the adaptation level had virtually reached a plateau after the first two sets. However, the adaptation levels significantly improved from the 2nd to the 3rd set only for the UBT group (*t*_(13)_ = 3.23, *p* = 0.039, corrected; Figure 6C). Thus, performing BM training after UM training contributed to improvement in the UM performance more than did continuance of UM training. The adaptation level of UM appeared to decrease from the 3rd to the 4th set, but this was not statistically significant (*t*_(13)_ = 2.02, *p* = 0.39, corrected).

Consistent with the result in the BT group, the BM adaptation level in the UT group was consistently smaller than the UM adaptation level (Figures 6A,B). In the UBT group, the training in the 3rd set was performed bimanually, which increased the BM adaptation level from the 2nd to the 3rd set. Indeed, a 2-way repeated measures ANOVA applied to the BM adaptation level revealed significant interactions between the UT and UBT groups (Groups and Sets: *F*_(1,3)_ = 21.42, *p* = 3.21 × 10^−10^, Figures 6A,B). There was a significant simple main effect of group, not for the 2nd set (*F*_(1,26)_ = 0.81, *p* = 0.37), but rather for the 3rd set (*F*_(1,26)_ = 22.59, *p* = 6.45 × 10^−5^). A significant simple main effect of sets was observed only for the UBT group (UBT group: *F*_(3,26)_ = 45.55, *p* = 1.72 × 10^−10^; UT group: *F*_(3,26)_ = 0.14, *p* = 0.93). Multiple comparisons with Bonferroni corrections indicated that the BM adaptation level remained unchanged after the 2nd set for the UT group (2nd and 3rd set: *t*_(13)_ = 0.087, *p* > 1, corrected), but for the UBT group, the adaptation level significantly increased from the 2nd to the 3rd set (*t*_(13)_ = 6.95, *p* = 6.08 × 10^−5^, corrected).

Figure 6D indicates that there was a significant correlation between the changes in UM performance from the 2nd to the 3rd set and those in BM performance (*R*^2^ = 0.688, *t*_(12)_ = 5.149, *p* = 2.415 × 10^−4^), again indicating that improvement in UM performance was likely to result from the increase in BM performance.

## Discussion

### Partially Overlapping Memory Structure of UM and BM

According to the partial motor learning transfer between UM and BM, we previously proposed the multi-compartment memory model, which consisted of three parts: UM-specific, BM-specific, and overlapping parts (Figure 1A; Nozaki et al., 2006; Nozaki and Scott, 2009). Consistent with these previous observations, the present study also provided evidence of partial learning transfer from UM to BM, and* vice versa*. For example, in the 2nd set for the BT and BUT groups, the ratio of UM adaptation level to BM adaptation level was 73.3 ± 3.3% (BT group) and 70.0 ± 4.6% (BUT group; Figures 4A,B). Similarly, in the 2nd set for the UT and UBT groups, the ratio of BM adaptation to UM adaptation level was 52.1 ± 6.1% (UT group) and 58.5 ± 6.6% (UBT) group (Figures 6A,B).

Previous, single-unit recording studies using nonhuman primates have demonstrated that the MI consists of neurons that respond specifically to either UM or BM (Donchin et al., 1998, 2002). For example, in the report by Donchin et al. (2002), among 187 primary motor cortex (MI) neurons recorded from two monkeys, 21 and 38 neurons were active for UM or BM, respectively, and 128 neurons were active for both types of movements. In other words, partially different neural populations in MI are involved in identical reaching actions between UM and BM. It is also well known that the neurons of MI change their activity patterns to adapt to a novel dynamic environment (Li et al., 2001; Arce et al., 2010). Consistent with these findings, the corticospinal excitability in humans while performing wrist movement, evaluated by transcranial magnetic stimulation to MI, also changed after the adaptation to a force-field (Kadota et al., 2014), indicating a significant role of MI for motor adaptation. Thus, partially different neuronal populations could be recruited for motor adaptation involving UM and BM, leading to development of a partially segregated memory structure.

### Improvement of BM Performance by Additional UM Training

The structure of partially segregated motor memories has been shown to enable identical reaching movements to adapt to conflicting force-fields, depending on whether the opposite arm is stationary (UM) or moving together (BM), which might contribute to flexible motor control under the presence of mechanical influence caused by movement of the opposite arm (Yokoi et al., 2011, 2014). However, this structure limits the transfer of the training effect from UM to BM. Although the UM practice is widely used and is considered useful for acquiring bimanual skills, this limiting factor of the training effect needs to be considered. In contrast to the intuitive idea of a limiting training effect, however, we speculated that, due to the memory structure, the UM training could improve the BM performance. This is because even after BM performance reaches a plateau, UM training could enhance BM performance by increasing the motor memory level in the overlapping part of the memory structure (Figure 1C).

Consistent with this speculation, we have demonstrated that interleaving UM training enhanced BM performance from the 2nd to the 3rd set (BUT group) more than did continuation of only BM training for the same number of trials (BT group; Figures 3A,B). We assumed that the beneficial effect of UM training was caused by an increment of the motor memory stored in the overlapping part of the memory structure (Figure 1C). This assumption was verified by the observation that the degree of improvement of BM was significantly correlated with that of UM induced by the interleaved UM training (Figure 4D).

An alternative explanation would be accounted for by the structure of the test period. For example, the test period of the 2nd set for the BUT group consisted of BM and UM error-clamp trials interleaved with BM training trials. Due to the decay of motor memory resulting from the error-clamp trials, the participants experienced relatively large movement errors during the BM training trials (Figures 3C,D), but no movement error during the BM error-clamp trials. Repeatedly experiencing two different errors for the same BM movements during the test period enabled the participants to detect the contextual change more easily, which might decrease the expression of motor memory (Vaswani and Shadmehr, 2013). In contrast, this decrease in BM memory might not occur in the test period of the 3rd set for the BUT group, because the participants performed only BM error-clamp trials. In other words, the BM motor memory was suppressed only when the BM error-clamp trials were interleaved with the BM training trials. However, this concept was unlikely to explain why the degree of improvement of BM was significantly correlated with that of UM induced by the interleaved UM training (Figure 4D). Furthermore, if this concept was correct, the movement error (i.e., lateral deviation of the handle) during the test period should have gradually increased within each set as the opportunity to experience two levels of movement error increased. However, we did not observe such a data trend in lateral deviation within each set (Figures 3C,D). Thus, our interpretation that the increase in BM memory resulted from UM training is more likely.

However, it should be noted that BM training in the 4th set did not improve UM performance (Figure 4B). This seems inconsistent with the beneficial effect of BM training on UM performance. However, as the BM performance had almost fully developed, virtually reaching a plateau by the end of the 3rd set, performing BM training in the 4th set could not add additional training effect to the overlapping memory structure. It should also be noted that the BM adaptation level appeared to become smaller from the 3rd to the 4th set, although the decrease was not significant. However, the BM adaptation level in the 4th set was not significantly different from that in the 2nd set, indicating that the beneficial effect of performing additional UM training on BM performance could be temporary. Future study is necessary to determine the duration of beneficial effect.

It should be noted that the nondominant left arm was used as the trained arm in the present study. Thus, one would wonder if the current idea is applicable when the dominant right arm is used as the trained arm. If the structure of the motor memory (Figure 1A) is different between right and left arms, the beneficial effect of UM training on BM performance should also change. The presence of different memory structures is possible, considering that sensorimotor areas in the dominant hemisphere have shown greater influence over the nondominant hemisphere in both functional magnetic resonance imaging (Hayashi et al., 2008; Diedrichsen et al., 2013) and electrophysiological studies (Netz et al., 1995; Oda and Moritani, 1995; Ziemann and Hallett, 2001; Duque et al., 2007).

In our previous study (Yokoi et al., 2014), we had right-handed participants adapt the forward movement of one arm (left or right) to a velocity-dependent curl force-field, while moving the opposite arm in the forward direction, and examined how this adaptation effect was influenced when the movement direction of the opposite arm was changed from the trained direction. We found that the influence on the left hand was greater when the movement direction of the right arm was changed, compared with the influence on the right arm from the left arm, indicating that the motor memory of the nondominant left arm is more strongly influenced by the movement pattern of the dominant right arm than vice versa. If the motor memory of the right arm is relatively independent of the movement of the left arm, as implied by the previous observation described above (Yokoi et al., 2014), the overlap between UM and BM for motor memory of the right arm should be greater than the overlap for motor memory of the left arm. Greater overlap should decrease the beneficial effect of UM training on BM performance, because it is obvious that the benefit is lost when the motor memories completely overlap. However, no previous work has systematically examined the laterality of the motor memory structure. Future work is necessary to address this issue and the resultant beneficial effect of UM training on BM performance for the dominant right arm.

### Influence of Memory Decay

We also need to consider how the memory stored in the BM-specific part changed during the UM training in the 3rd set of BUT group. Since the BUT group performed only UM training during the 3rd set, the memory stored in the BM-specific part of the memory structure was not updated during the 3rd period. This should result in time-dependent memory decay. If the amount of memory decay in the BM-specific part was greater than the increment in the overlapping part induced by the UM training, the total amount of motor memory for BM (i.e., stored in BM-specific and overlapping parts) would decrease.

**Figure 7:**
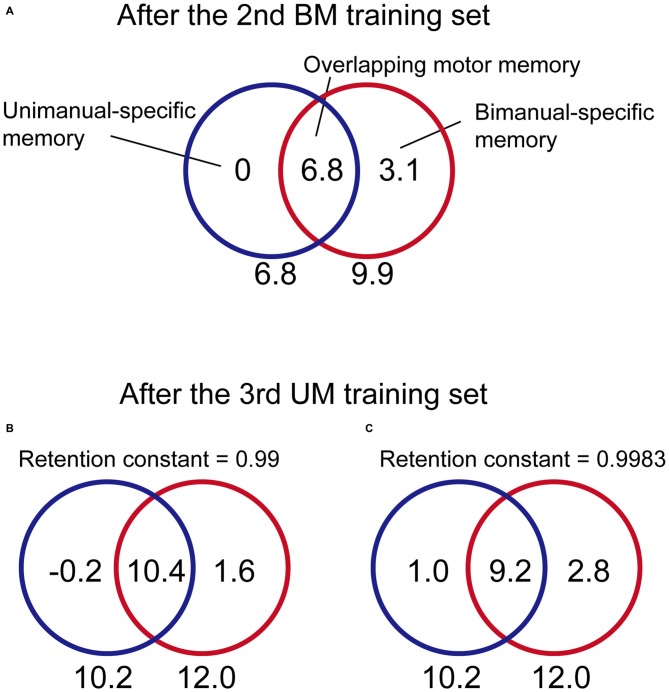
**Estimation of how the content for each memory part changed from the 2nd to the 3rd training set for the BUT group. (A)** From the data after the 2nd BM training for the BUT group (the adaptation level was 9.9 and 6.8 for BM and UM, respectively; see Figure 4B), the content for each memory part can be estimated as 0 (UM-specific part), 6.8 (overlapping part), and 3.3 (BM-specific part).** (B)** During the subsequent 3rd UM training, the memory in the BM-specific part was not updated, but should decay with time. If the retention constant was assumed to be 0.99, according to our previous work (Nozaki and Scott, 2009), then the memory content for the BM-specific part should decrease to 1.6. Using the data from after the 3rd UM training (the adaptation level was 12.0 and 10.2 for BM and UM, respectively; see Figure 4B), the memory content could be estimated at −0.2 (UM-specific part) and 10.4 (overlapping part). However, this does not seem possible, because this indicates that the memory content in the UM-specific part decreased to a negative value with UM training. **(C)** In contrast, if the retention constant was greater, as proposed by Ingram et al. (2013); we could estimate that a reasonable amount of memory content was to be found in all three parts. See the main text for the details of estimation.

We investigated our data from this point of view. In the BUT group, after the 2nd set, the after-effect of BM and UM was 9.9 and 6.8, respectively (Figure 4B), indicating that the amount of memory in the overlapping part was 6.8 and that in the BM-specific part was 3.1 (= 9.9–6.8; Figure 7A). In the model we previously proposed (Nozaki and Scott, 2009), the memory stored in the BM-specific part while performing UM was assumed to decay with a constant of 0.99 with every UM trial. According to this assumption, the memory in the BM-specific part should decrease to 53% (= 0.99^64^) during the UM training trials in the 3rd set in the BUT group. Thus, the amount of memory stored in the BM-specific part can be estimated as 3.1 × 0.53 = 1.6 (Figure 7B).

On the other hand, after the UM training in the 3rd set of the BUT group, the after-effect of BM and UM was 12 and 10.2, respectively (Figure 4). Assuming that the memory in the BM-specific part is 1.6, as calculated above, the amount of memory in the overlapping part should be 10.4 (= 12 − 1.6; Figure 7B). However, this is not possible, as the UM after-effect indicates that the memory in the overlapping part cannot exceed 10.2 (Figure 7B). Therefore, if the memory in the BM-specific part decays by a constant of 0.99 with every trial, the increase in the motor memory in the overlapping part by UM training cannot compensate for the decrement of the memory in the BM-specific part.

How then is the beneficial effect explained? Recently, it has been reported that the decay of motor memory is context-dependent (Ingram et al., 2013). In this study, after adapting reaching movements in the backward direction to the velocity-dependent force-field, participants performed 30 error-clamp reaching trials in a backward direction (i.e., in the same context) or in a forward direction (i.e., in a different context). During the error clamp trials, the adaptation level of reaching movement in backward direction decayed, but the amount of decay was smaller after 30 reaching movements in the forward direction than in the backward direction. Therefore, they concluded that the decay of motor memory was smaller when performing movements that were different from the training movement.

We assumed that this context-dependent decay effect was also likely to occur during UM and BM. More specifically, when the participants performed UM training, the motor memory in the BM-specific part decayed only slightly. When the retention constant was assumed to be 0.9983, as reported by Ingram et al. (2013), the amount of memory stored in the BM-specific part became 3.1 × 0.9983^64^ = 2.8 by the end of UM training (Figure 7C). The memory in the overlapping part can be estimated to be 9.2 (= 12–2.8). This value indicates that the memory stored in the overlapping part increased by 2.4 (from 6.8 to 9.2). Considering the UM after-effects after the UM training (i.e., 10.2), the memory in the UM-specific part should be 1.0 (= 10.2 − 9.2). Thus, the UM training increased memory in the UM-specific part and the overlapping part from the 2nd to the 3rd set by 1.0 and 2.4, respectively, indicating that the beneficial effect of UM training on BM performance can be explained by assuming that the memory in BM-specific part remains stable during UM training. The beneficial effect of BM training on UM performance can be explained in the same way.

### Practical Implications

The issue of how effectively and rapidly motor skills can be acquired (or reacquired) is important for athletes, musicians, and patients with motor dysfunction. For bimanual motor skills, one of the strategies commonly adopted is to break down a whole motor skill into simpler unimanual skills, and then train each hand (or arm) separately (part practice; McArthur, 1997; Burke, 2002; Finch, 2004; Schmidt and Wrisberg, 2007; Schmidt and Lee, 2011). Intuitively, this type of practice should be beneficial for acquiring motor skills by each hand (or arm) before doing it bimanually, but this had not been sufficiently investigated until we had previously demonstrated that the training effect of UM practice was only partially transferred to the same movement performed bimanually (Nozaki et al., 2006). The results of this study seemed to imply that the effect of UM training on BM performance was limited, but the present study has demonstrated that this is not the case: we show that BM performance can be facilitated by performing UM training. As shown in Figures 4B,C, even if the BM performance had reached a plateau, subsequently performed UM training could improve the BM performance, possibly through an increase in the motor memory content of the overlapping part of the memory structure. This result may provide a novel insight into the reason for the efficacy of UM training in improving BM performance.

Thus far, we have emphasized the beneficial effect of UM training on BM performance; however, based on the consideration of motor memory structure for UM and BM (Figure 1A), we also expected a beneficial effect of BM training on UM performance. Indeed, we verified that BM training after sufficient UM training may also facilitate UM performance. The beneficial effect of BM on UM performance has been reported for the bilateral training adopted for rehabilitation in stroke patients (Cunningham et al., 2002; Cauraugh and Summers, 2005; Choo et al., 2015). The effect of bilateral training on the paretic arm is thought to be because simultaneous movement of the nonparetic limb can facilitate movement of the paretic limb through the interlimb coupling observed in healthy individuals (Swinnen, 2002). Our study has provided further insights into why BM training is beneficial for improving UM performance. To our knowledge, except for the bilateral training described above, no previous studies have shown the beneficial effect of BM training on UM performance. Considering that sports using only one arm (e.g., tennis, throwing a ball, etc.) are ubiquitous, it would be of practical interest to investigate how training with moving the opposite arm could improve UM motor skills.

These findings also indicate that the beneficial effect is not related to the notion that UM is a part of BM, but is related to the overlapping memory structure. Recent studies have shown that the motor memories for identical (reaching) movements are changed according to different behavioral contexts, such as the movement pattern of the opposite arm (Yokoi et al., 2011, 2014), discrete vs. rhythmic movements (Ikegami et al., 2010; Howard et al., 2011), or the movement pattern of the lead-in or follow-through movements (Howard et al., 2015). It should be noted that motor memories for different contexts are not completely distinct, but overlap partially. The presence of an overlapping memory structure suggests that performing training in different behavioral contexts rather than in the original context might help to facilitate motor skill development because it might increase the memory content in the overlapping part of the memory structure. This concept is consistent with the classical idea that variable training is more effective for acquiring motor skills (McCracken and Stelmach, 1977; Shea and Kohl, 1991). However, this concept was based on the schema theory (Schmidt, 1975), according to which variable training could contribute to developing a generalized motor program. However, our study provides another mechanism by which variable practice can have a beneficial effect. Finally, since the present study focused only on the short-term training effect, it is not clear how long the facilitated training effect would last. Future studies are necessary to examine the long-term effect of performing training in different behavioral contexts.

## Author Contributions

TH performed the experiments and analyzed the data. TH and DN designed the study and wrote the manuscript.

## Conflict of Interest Statement

The authors declare that the research was conducted in the absence of any commercial or financial relationships that could be construed as a potential conflict of interest.
